# Dynamic magnetic resonance imaging in assessing lung function in adolescent idiopathic scoliosis: a pilot study of comparison before and after posterior spinal fusion

**DOI:** 10.1186/1749-799X-2-20

**Published:** 2007-11-19

**Authors:** Winnie CW Chu, Bobby KW Ng, Albert M Li, Tsz-ping Lam, Wynnie WM Lam, Jack CY Cheng

**Affiliations:** 1 Departments of Diagnostic Radiology and Organ Imaging, The Chinese University of Hong Kong, Prince of Wales Hospital, Shatin, Hong Kong, China; 2 Departments of Orthopaedics and Traumatology, The Chinese University of Hong Kong, Prince of Wales Hospital, Shatin, Hong Kong, China; 3 Departments of Paediatrics, The Chinese University of Hong Kong, Prince of Wales Hospital, Shatin, Hong Kong, China

## Abstract

**Background:**

Restrictive impairment is the commonest reported pulmonary deficit in AIS, which improves following surgical operation. However, exact mechanism of how improvement is brought about is unknown. Dynamic fast breath-hold (BH)-MR imaging is a recent advance which provides direct quantitative visual assessment of pulmonary function. By using above technique, change in lung volume, chest wall and diaphragmatic motion in AIS patients before and six months after posterior spinal fusion surgery were measured.

**Methods:**

16 patients with severe right-sided predominant thoracic scoliosis (standing Cobb's angle 50° -82°, mean 60°) received posterior spinal fusion without thoracoplasty were recruited into this study. BH-MR sequences were used to obtain coronal images of the whole chest during full inspiration and expiration. The following measurements were assessed: (1) inspiratory, expiratory and change in lung volume; (2) change in anteroposterior (AP) and transverse (TS) diameter of the chest wall at two levels: carina and apex (3) change in diaphragmatic heights. The changes in parameters before and after operation were compared using Wilcoxon signed ranks test. Patients were also asked to score their breathing effort before and after operation using a scale of 1–9 with ascending order of effort. The degree of spinal surgical correction at three planes was also assessed by reformatted MR images and correction rate of Cobb's angle was calculated.

**Results:**

The individual or total inspiratory and expiratory volume showed slight but insignificant increase after operation. There was significantly increase in bilateral TS chest wall movement at carina level and increase in bilateral diaphragmatic movements between inspiration and expiration. The AP chest wall movements, however, did not significantly change.

The median breathing effort after operation was lower than that before operation (p < 0.05).

There was significant reduction in coronal Cobb's angle after operation but the change in sagittal and axial angle at scoliosis apex was not significant.

**Conclusion:**

There is improvement of lateral chest wall and diaphragmatic motions in AIS patients six months after posterior spinal fusion, associated with subjective symptomatic improvement. Lung volumes however, do not significantly change after operation. BH-MR is novel non-invasive method for long term post operative assessment of pulmonary function in AIS patients.

## Background

Adolescent idiopathic scoliosis (AIS) is the most common form of idiopathic scoliosis, typically affecting growing adolescent girls, 10–16 years of age. Untreated scoliosis has an increased risk of developing respiratory failure and premature mortality[1]. Pulmonary function impairment in AIS patients might be related to restricted lung volume, poor chest wall expansibility or impaired diaphragmatic motion. Little is known about which of the above factors is more significantly correlated with the pulmonary deficit in AIS. Restrictive impairment is the commonest reported pulmonary deficit in AIS, which improves following surgical operation[2,3]. However, the exact mechanism of how the improvement is brought about is unknown.

We have previously reported a validated novel imaging technique for assessment of pulmonary function in AIS subjects. Kotani and colleagues have also investigated on chest wall and diaphragmatic movement of scoliosis patients using dynamic breathing MRI [4]. With the application of ultrafast dynamic breath-hold (BH) MR imaging and multiplanar reformat technique, the lung volume, chest wall, and diaphragmatic motions between inspiration and expiration can be accurately measured with high reproducibility in both AIS subjects and normal controls[5].

The aim of this pilot study was to evaluate the change in lung volume, chest wall and diaphragmatic motion in AIS patients before operation and six months after posterior spinal fusion surgery.

## Methods

### Subjects

The study included 16 idiopathic scoliosis girls with a predominant right-sided thoracic curve (Standing Cobb's angle ranged from 50°–82°, mean 60°). The distribution of curves by Lenke classification is given in Table 1. Their age ranged from 11–18 (mean 14.4). They were consecutively included for scheduled posterior spinal fusion without thoracoplasty from 2002 to 2004. The surgical procedure was performed under SSEP monitoring. It consisted of a standard midline posterior exposure, subperiosteal dissection made from spinous process to the tips of each transverse process. The facet capsules were meticulously elevated till the superior rib surface was reached. Partial excision of the ligamentum flavum was made between spinous processes for the lordotic thoracic segments typically from T5 to T11. The exposure was made to preserve vascularity to paraspinal muscles. Instrumentation consisted of three types. (a) 3 cases of Harrington rod on concave side, Luque rod on convex side and Wisconsin wire for the instrumented spinous processes. (b) 8 cases of ISOLA instrumentation consisted of pedicle screws to lumbar segments to build a base, proximal claw hook construct at two segments above upper end vertebra on both sides followed by pedicle hook at upper end vertebra on the concave side and transverse process hook on convex side. Wisconsin wires were placed for the mid-thoracic segments. (c) 5 cases of CDM8 instrumentation were essentially same as the ISOLA constructs except the lumbar pedicle screws used were top loading monoaxial screws instead of vertebral screws connecting to rod with slotted connectors as in the ISOLA system. After placement of the fixation devices, curve reduction was made by manual pressure at apex and counter pressures at opposite ends under SSEP monitoring. Rod estimation and preliminary contouring was then made. Decortication of facet joints and transverse processes was made and bone grafts were placed at each decorticated facet joint. Instrumentation was then performed and final rod contouring made with insitu benders. Cell saver was used to retrieve blood and transfused back to patient intra-operatively. There were no neurological or wound complications. All AIS subjects were neurologically normal on detail clinical examination. Exclusion criteria included history of back injury, weakness or numbness in one or more limbs, urinary incontinence or nocturnal enuresis. None of the subjects had any history of pulmonary diseases and they were free from any respiratory symptoms or acute respiratory infection at the time of the MR studies before and after operation.

**Table 1:** Frequency of curve types by Lenke classification in 16 subjects included in this study

Lenke's classification	Frequency
1A-	1
1AN	6
1BN	5
1C-	1
2A-	1
3AN	1
3BN	1

Ethical approval and informed consent for dynamic breath-hold MR imaging have been obtained from all the subjects and their parents.

### MRI assessment

Pre operative MRI examination of the chest was performed in all subjects within two weeks before the operation, while the post operative MRI was performed after 6 months from the date of surgery. MRI examination was performed using a 1.5T MR scanner (Sonata, Siemens, Erlangen, Germany). The protocol of BH-MR imaging of the chest has been reported in previous published study [5]. In brief, fast gradient-recalled echo pulse sequence was used to obtain coronal images of the whole chest during full inspiration and expiration with parameters. The images were acquired in the supine position. All subjects were given clear instruction by experienced radiographers and practice took place before actual imaging. The subjects were instructed to fully inspire/fully expire and then hold their breaths at either full inspiration or full expiration. Scanning repeated three times for full inspiration and another three times for full expiration. Each breath-hold scan took about 15–20 seconds, which was well tolerated by all subjects. The images with the maximum inspiratory and expiratory effort out of the three attempts were chosen for analysis.

**Figure 1:**
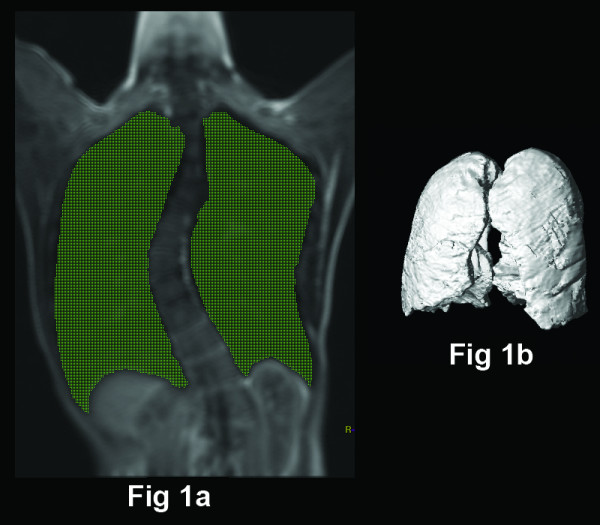
Measurement of lung volumes by a semi-automated computerized method of delineating the lungs and summing cross-sectional areas. (a) On the coronal image of the lung, threshold signal intensity is selected to highlight the air in green (lung). (b) The total lung volume is calculated by summating the volume of all coronal sections of the lungs from the front to the back of the body.

**Figure 2:**
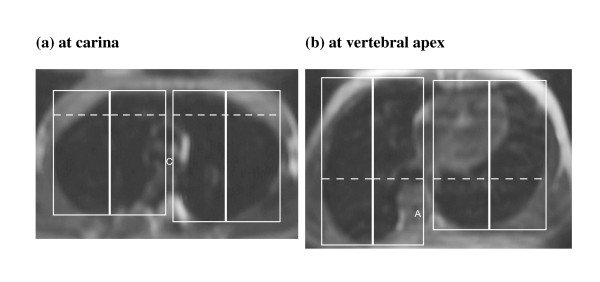
Measurement of AP and TS diameter of the chest wall on the reformatted axial image. (a) Upper level at the carina (C), maximal inspiratory image. (b) Lower level at the apical vertebra (A), maximal inspiratory image. Tangential lines are drawn to the anterior, posterior and lateral lung surfaces. The chest wall dimensions are then measured as the largest anteroposterior (AP, thick solid lines) and transverse (TS, dotted lines) dimensions on either side of the scoliosis separately. The chest wall motion is calculated as the difference between inspiration and expiration.

**Figure 3:**
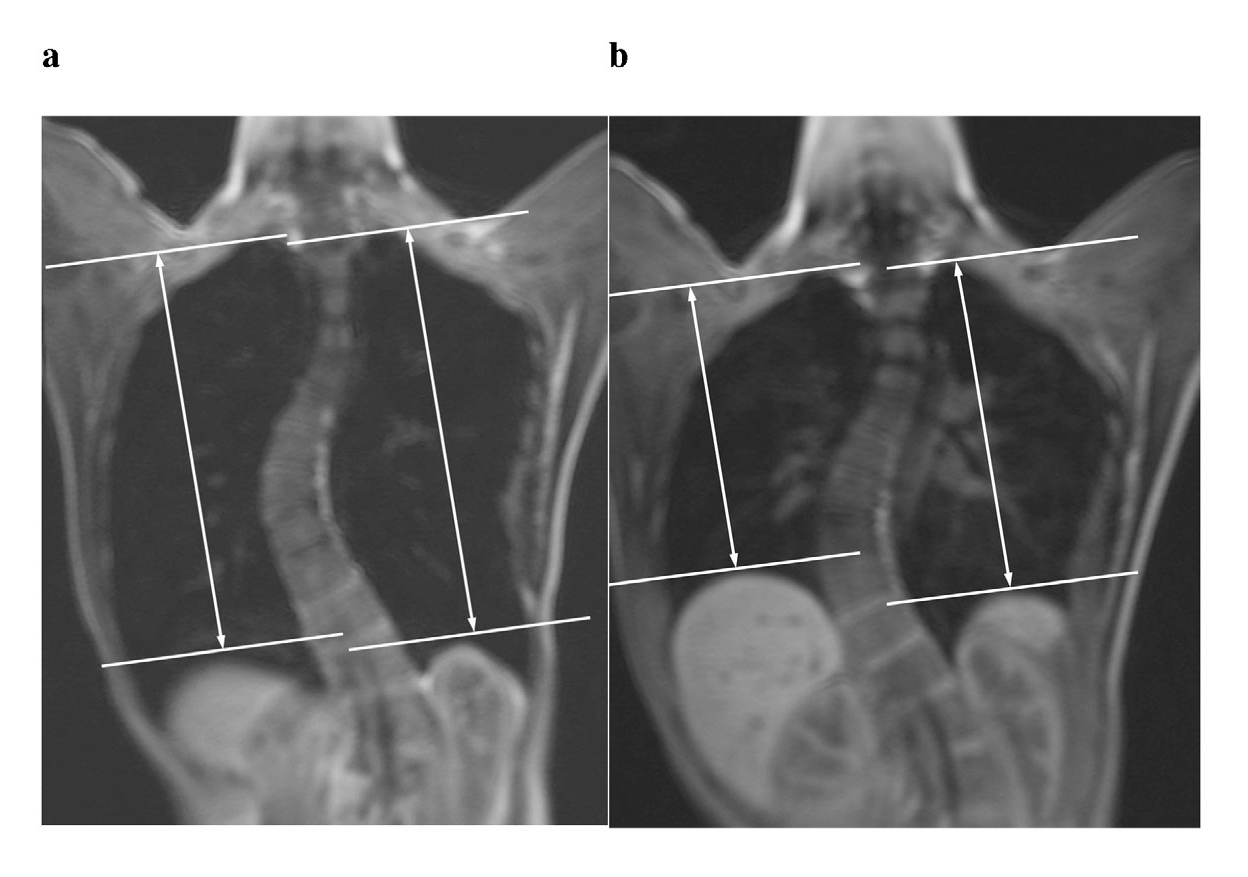
Measurement of diaphragmatic heights on the reformatted coronal image. (a) At maximal inspiratory image and (b) maximal expiratory image The diaphragmatic heights were taken as the vertical distance in between the line drawn tangent to the highest point of the diaphragm and a parallel line to the lung apex. The diaphragmatic motion is calculated as the difference between inspiration and expiration.

Post-processing of the MR images was performed using a workstation (EasyVision, Philips Medical Systems, Best, the Netherlands). Volumetric measurements of total inspiratory and expiratory lung volume were determined by a semi-automated computerized segmentation method [6] (Fig 1). The MR images were also reformatted into axial and coronal planes so that motions of the chest wall and diaphragm could be assessed. The chest wall and diaphragmatic motions were measured in antero-posterior, left-right and cranio-caudal directions respectively. The chest wall diameters were measured at the level of the carina (Figure 2a) and at apex of the vertebral curve (Figure 2b) respectively. The chest wall dimensions were then measured as the largest anteroposterior (AP) and transverse (TS) dimensions on either side of the scoliosis separately. The diaphragmatic heights were taken as the vertical distance between the line drawn tangential to the highest point of the diaphragm and a line parallel to the lung apex (Fig 3a and 3b). All the lung volume, chest wall and diaphragmatic dimensions in the right and left hemithorax were measured separately, during both inspiration and expiration, and the differences were recorded.

Three measurements were made for each parameter by the same observer and the average value was taken for the analysis. Our previous studies showed high intraobserver and interobserver reliability on the MR measurements [5].

### Breathing Effort Assessment

Patients were also asked to score their breathing effort before and after operation using a scale of 1–9 with ascending order of effort. Score 0 was equivalent to non-awareness of breathing effort at rest. Score 1–3 was equivalent of awareness of gentle breathing effort at rest with ascending order of effort. Score 4–6 was equivalent to feeling of breathlessness during exercise activities in ascending order of severity. Score 7–9 was equivalent to feeling of breathlessness at gentle exertion such as walking in ascending order of severity.

### Statistics

The parameters in each group of subjects were expressed as median with inter-quartile range. Non-parametric Wilcoxon signed ranks test was used to compare the MR measurements before and after operation in all subjects.

Two-tailed probability values <0.05 were considered significant. SPSS for Windows statistical software (Release 13, SPSS Inc., Chicago, Illinois) was used in the analysis.

## Results

The spatial resolution of the reformatted axial and coronal images of the chest was considered to be of diagnostic quality in all subjects. The fast image acquisition time allows good delineation of the lung volume, the chest wall and the diaphragm.

Surgical procedures in all patients were uneventful without significant complications. All patients were discharged home fully mobilized on average 11 days (range 9–15) from admission. Six month after operation, the median correction rate of Cobb's angle was 60% (range 42% to 72%). The median Cobb's angle changed from 59° to 24° while the sagittal Cobb's angle changed from 19.1° to 23.1°.

The lung volumes, chest wall and diaphragmatic parameters before and after operation in all subjects are summarized in Table 2.

**Table 2:** Lung, chest wall and diaphragmatic parameters, spinal curvatures in 16 subjects before and six months after corrective posterior spinal fusion. Figures are expressed as median (interquartile range)

	Before surgery	After surgery	P value (* significant at 0.05 level)
Inspiratory volume (cc)			
Right lung	1719 (1232, 1858)	1764 (1362, 1938)	0.57
Left lung	1392 (1113, 1607)	1381 (1152, 1642)	0.35
Total	3127(2364, 3464)	3152 (2491, 3529)	0.50

Expiratory volume (cc)			
Right lung	662 (579, 817)	716 (586, 814)	0.61
Left lung	598 (501, 700)	601 (464, 707)	0.96
Total	1269 (1083, 1498)	1361 (1038, 1477)	0.64

Change in lung volume (cc)			
Right lung	996 (645, 1126)	969 (698, 1167)	0.68
Left lung	762 (535, 962)	736 (630, 1018)	0.22
Total	1770 (1206, 2089)	1730 (1362, 2203)	0.30

Right lung AP diameter at carina (mm)			
Inspiratory	117 (100, 123)	118 (107, 130)	0.07
Expiratory	84 (75, 92)	88 (82, 96)	0.06
Change	29 (21, 42)	29 (21, 42)	0.98

Left lung AP diameter at carina (mm)			
Inspiratory	122 (106, 133)	119 (108, 129)	0.18
Expiratory	89 (80,99)	93 (87, 101)	0.41
Change	29 (16, 46)	27 (18,33)	0.14

Right lung AP diameter at apex (mm)			
Inspiratory	135 (123, 151)	137 (116, 155)	0.50
Expiratory	1114 (99, 126)	114 (100, 133)	0.88
Change	21 (15,31)	20 (13, 25)	0.20

Left lung AP diameter at apex (mm)			
Inspiratory	126 (118, 145)	126 (121, 138)	0.92
Expiratory	104 (94, 114)	104 (97, 111)	0.72
Change	28 (17, 37)	23 (12, 33)	0.30

Right lung TS diameter at carina (mm)			
Inspiratory	97 (80, 116)	111 (106, 116)	0.013*
Expiratory	85 (75, 93)	87 (75, 96)	0.30
Change	15 (3, 27)	28 (17, 33)	0.006*

Left lung TS diameter at carina (mm)			
Inspiratory	92 (79, 103)	104 (99, 110)	0.002*
Expiratory	79 (59, 84)	86 (76, 93)	0.044*
Change	14 (11, 23)	20 (13, 28)	0.056

Right lung TS diameter at apex (mm)			
Inspiratory	106 (85, 126)	114 (105, 120)	0.039*
Expiratory	87 (71, 92)	91 (84, 100)	0.034*
Change	16 (10, 31)	24 (13, 32)	0.796

Left lung TS diameter at apex (mm)			
Inspiratory	112 (93, 117)	113 (104, 122)	0.088
Expiratory	98 (68, 113)	100 (83, 112)	0.163
Change	9 (6, 13)	12 (7, 17)	0.301

Right Diaphragmatic height (mm)			
Inspiratory	185 (172, 202)	201 (182, 223)	<0.01*
Expiratory	136 (129, 150)	146 (135, 156)	0.196
Change	49 (25, 65)	53 (44, 66)	0.039*

Left Diaphragmatic height (mm)			
Inspiratory	185 (172, 206)	198 (186, 215)	0.01*
Expiratory	142 (129, 159)	148 (136, 161)	0.234
Change	44 (33, 57)	53 (44, 66)	0.039*

Breathing effort	4 (1,4)	1.75 (1, 3)	0.012*
Coronal Cobb angle (MR)	59 (53, 64)	24 (21, 28)	<0.01*
Axial angle (MR)	9.9 (4.7, 13.0)	9.6 (4.0, 14.7)	0.215
Sagittal angle (MR)	19.1 (13.1, 28.8)	23.1 (17.2, 31.2)	0.056

For lung volumes, the right lung (on the convexity side of the scoliotic curve) and total inspiratory and expiratory lung volume showed slight but insignificant increase after operation. Neither right/left lung nor the total vital lung capacity (defined as the difference in lung volume between inspiration and expiration) differ significantly when comparing the pre-operative with the post-operative study.

For the TS diameter of bilateral chest wall, there was significant increase in baseline value on both right and left lung at either carina level or apical vertebral level during both inspiration and expiration. When the difference between inspiration and expiration was considered, the lateral chest wall movement at carina level was improved significantly on right/convex side (p = 0.013) while marginally significant on left/concave side (p = 0.056). but there was no statistically significant change at the apical vertebra level.

For the AP diameter of bilateral chest wall, there was no significant difference on either right lung or left lung in between operation. The AP chest wall movement also showed no significant interval change.

For the diaphragmatic heights, they were significantly increased on both right and left sides during inspiration while the absolute value during expiration was unchanged and hence the motion of the diaphragms, defined as the difference in diaphragmatic height between inspiration and expiration, was significantly increased after operation when compared with the pre-operative stage.

The median breathing effort reported by the subjects before operation was 4 (range 1–9) while the median breathing effort after operation was 1.75 (range 1–5).

In summary, there was improvement of lateral chest wall and diaphragmatic motions in AIS subjects six months after posterior spinal fusion. Lung volumes however, did not show significant increase after operation at this stage.

## Discussion

Though pulmonary function impairment has long been reported in AIS patients, it is difficult to measure and describe respiratory motions because of the limited methods available for real time dynamic assessment. In the present and previous published study, we have demonstrated that by using multi-planar reformat technique, coronal and axial sectional planes could be obtained simultaneously with measurement of lung volumes during a single inspiration and expiration movement. This MR technique has also been validated which showed significant positive correlations with plethysmography parameters[6]. We therefore propose the use of dynamic breath-hold MR as a novel non-invasive tool for clinical analysis of lung volume, diaphragmatic and chest wall motion in AIS patients.

In this study, dynamic BH-MR imaging has been used to compare the pre and post operative lung function changes in AIS patients after spinal fusion. We found that the metallic implants only caused mild distortion artefacts on the immediate adjacent structures such as the vertebral column and the central canal; while the visualization of the lungs, the chest walls and diaphragms were not affected.

In the literature, there has been debate about the effect of surgical correction on lung function. Many authors have written that scoliosis correction definitely improves measured pulmonary functions [7], such as vital capacity. Others disagree and declare that pulmonary functions remain essentially unchanged [8-10] or even becoming worsen[11].

In the previous published study, we found that the chest wall and diaphragmatic motion in AIS patients were not restricted, i.e. the chest wall and the diaphragmatic motions were as mobile as those in the normal subjects and therefore there was no suggestion of neuromuscular dysfunction in AIS [5].

In the current study, we found that the chest wall motion and diaphragmatic motions could be further improved after posterior spinal surgery. This was consistent with the subjective feeling of reducing breathing effort after operation as reported in this group of patients. The reason for increased amplitude of lateral chest wall movement could be explained by the less distorted chest wall configuration after surgery, while the improvement of diaphragmatic excursion could be the combined effect of chest wall remodeling, lessen compressive effect by both the scoliotic spine and the rotated mediastinum as well as improvement in degree of hypokyphosis after surgery.

The absolute lung volumes however, did not show significant increase in this cohort. As the current study was carried out shortly after the operation within six months, longer follow up study is warranted which might show a change in pulmonary volume.

Dynamic MR is considered as a promising investigation tool in assessing respiratory mechanism in the AIS group. It might be useful for both short and long term follow up of pulmonary function of AIS patients, in particular, for assessing post operative changes.

## Conclusion

With the application of ultrafast dynamic BH-MR imaging and multi-planar reformat technique, the lung volume, chest wall and diaphragmatic motion between inspiration and expiration could now be accurately measured with high reproducibility in AIS patients. Improvement of lateral chest wall and diaphragmatic motions are evident in AIS patients six months after posterior spinal fusion. BH-MR might be sued for long term post operative assessment of pulmonary function in AIS patients.

## Abbreviations

AIS: Adolescent Idiopathic Scoliosis

BH: breath-hold

MR: Magnetic Resonance

## Competing interests

The author(s) declare that they have no competing interests.

## Authors' contributions

All the authors have contributed to conception and design of the manuscript. Particularly, WCWC and BKWN carried out the analysis of data and wrote the manuscript; AML and TPL contributed to the editing of the manuscript, clinical recruitment and assessment; WWML participated in the editing of the manuscript; JCYC contributed to the editing of the manuscript and secured funding. All authors have read and approved the final manuscript.
